# Whether and when disclosing the trauma to one’s children in a migratory context? A pilot mixed methods investigation

**DOI:** 10.1186/s40359-022-00858-w

**Published:** 2022-06-07

**Authors:** Elodie Gaëlle Ngameni, Mayssa’ El Husseini, Elisabetta Dozio, Cyrille Kossigan Kokou-Kpolou, Gisèle Apter, Marie Rose Moro

**Affiliations:** 1 Universite Sorbonne Paris Nord, 93430 Villetaneuse, France; 2 Maison de Solenn-Maison of Cochin’s Youth, APHP, 75014 Paris, France; 3 University of Picardie Jules Verne CHSSC EA 4289, Amiens, France; 4 Mental Health and Care Practices, Gender and Protection Sector, Action Contre la Faim (ACF), Paris, France; 5 Faculty of Social Sciences, School of Psychology, Laval University, Quebec City, Canada; 6 Group Hospitaller of Havre and University of Rouen Normandie, Rouen, France; 7 Department of Maison de Solenn Maison of Cochin’s Youth, APHP, 75014 Paris, France; 8 University of Paris, PCPP, 92100 Boulogne-Billancourt, France; 9 CESP, Faculty of Medicine, University Paris South, Paris, France; 10 Faculty of Medicine—UVSQ, Inserm, University of Paris-Saclay, 94805 Villejuif, France

**Keywords:** Disclosure, Trauma narrative, Mother–child, Transmission, Migration

## Abstract

**Background:**

Disclosing traumatic events experienced by parents to their children is a central issue in the intergenerational trauma transmission. However, little is known about this question among migrant population. The main objective of this study was to examine the choice to disclose the traumatic experiences of migrant women in France to their children.

**Methods:**

This pilot study examined fourteen mother–child dyads in which migrant mothers (M = 30 years; range = 19–42 years) were exposed to traumatic events. A sequential mixed method design was used. In addition to the completion of the Impact Event Scale-Revised, qualitative data were collected through semi-structured interviews. These data were analyzed using thematic and cross-cultural methods. The survey took place from May 2019 to July 2020.

**Results:**

Our study revealed three profiles of mothers with regard to the choice to disclose the traumatic story to the child: one group of mothers opted for silence (n = 4), the other for disclosure (n = 7) and the last group who were hesitant (n = 3). The modalities of choice were statistically associated with the severity of the post-traumatic stress symptoms, F (2, 11) = 4,62, p < .05. Specifically, women who made the choice of silence (M = 72.75, SD = 4.99) and those hesitated on the choice to disclosure (M = 71.33, SD = 7.51) reported higher scores on IES-R than those who made the choice to disclosure (M = 59.86, SD = 12.44). Six main themes emerged from the thematic and cross-cultural analysis of participants’ narratives: (1) the personalization of the traumatic experience, (2) the child seen as a weapon against collapse, (3) the fear of the child's personal reactions, (4) the possible partial disclosure, (5) the trauma narrative according to the child's age, and (6) the trap of the in-between two cultures.

**Conclusion:**

Our results suggest that the recovery of these mothers from their trauma, through culturally appropriate therapeutic care, can effectively contribute to the choice to disclose their traumatic experiences to their children. This treatment can support them in developing open and healthy communication strategies to prevent the transmission of traumatic effects to their children.

## Introduction

Should the parents share or keep silent the traumatic events they experienced to their offspring children? When should it be told and in what context? Depending on the theoretical framework, researchers have reached different conclusions on these questions. For some researchers, silence is seen as a protective factor for the child. Studies carried out among Bosnian and Middle Eastern families and refugee children in Sweden and Denmark respectively indicate a negative impact of open communication. Specifically, the parents' communicating of their traumatic experiences seemed to exacerbate the negative emotions of their children [1, 2]. However, other authors note that non-disclosure of the traumatic story, or silence within families, is associated with the intergenerational transmission of traumatic suffering. This phenomenon, known as the "conspiracy of silence", has been observed in the families of Holocaust survivors [3, 4]. Psychodynamic theorists have also shown that a parent who has experienced a major trauma, and who has not communicated openly with his or her children, would transmit the trauma to the children in the form of unconscious displaced emotion [5, 6].

For some researchers, there is a need for an open and a healthy communication of traumatic experience. This sharing of traumatic experiences from parents to their children is seen as a process that promotes the reconstruction of traumatic identities [3, 4, 7, 8]. Braga et al.'s qualitative study of descendants of Holocaust survivors living in Brazil shows the impact of trauma depending on the ways in which survivors communicate the traumatic message to their offspring [3]. When the modes of communication of survivors are permeated by secrets, silences and unspoken words, the impact on offspring is manifested in a terrifying worldview, experiences of guilt, victimisation and submission. In contrast, open communication of parents' traumatic experiences to their children is the source of the use of humour as a symbolic resource. This model of open communication has also been observed as a source of artistic creation and appropriation of parental resilience models.

Still other authors argue that communication is necessary, but it should be done in a way that takes into account the circumstances, the age, and the way in which the traumatic experience is to be expressed to the children [9–12]. Rousseau et al. have put forward the notion of 'modulated disclosure' in which parental sensitivity to the child's cognitive and emotional needs is more important than the content of what is disclosed [12]. These authors note the importance of the temporality of disclosure and how the story is told rather than the effects of open or silent communication [9]. Furthermore, from a social and constructivist perspective, De Haene et al. show that communication is necessary, but that it must take into account the contexts and cultures of the families [13]. Their research in Belgium and Denmark with migrant families and their children of about 4–9 years emphasises the importance of affective and open parental communication about traumatic elements in order to support children's resilient capacities to cope with transmitted war-related trauma.

These aforesaid studies, whether conducted among refugees who have left their countries of origin at war or among people who have experienced collective trauma such as the Holocaust, involved families and especially first, second and third generation children about the type of communication used by their parents [3, 11, 12]. These studies, and more specifically the review of the literature by Dalgaard and Montgomery [9], revealed that the type of communication used by parents about their traumatic experience had negative consequences on the psychological development of their children. No study in France has hitherto examined the communication of migrant parents' traumatic experiences to their children. We were interested in the nature of the trauma narrative of migrant women in France. The particularity of the present study is to examine the implications of the disclosure of the traumatic story experienced in a migratory context, from the mother to the child. How do these mothers project themselves into the disclosure of the traumatic event to their children? Using a mixed method design, we seek to answer three main questions:

1. Does the choice of mothers to disclose or not their children the story of the trauma depends on the type of trauma experienced?
2. Does this choice depend on the severity of the post-traumatic stress symptoms?
3. What are the motivating factors for this choice that emerge from the women’s narratives?

## Methods

### Methodological design and ethics

We adopted a sequential mixed method design, given the exploratory nature of the present study [14, 15]. Participants were requested to complete the Impact of Event Scale-Revised 10 (IES-R) [16]. The total IES-R scores allowed to determine the severity of the post-traumatic symptoms of each participant. Additional qualitative data through semi-structured interviews were collected and analysed to develop a deeper understanding of the quantitative data. The research protocol has been validated by the ethics committee of the research laboratory (no. 12-065) and has been accepted within the hospital group.

### Data collection procedures

The study was conducted in France. The field survey took place from May 2019 to July 2020 and covered a period of 15 months. The recruitment of the participants was carried out within the child psychiatry department, the mother-baby care unit of the Hospital Group. This medical and psychological consultation unit is a multidisciplinary perinatal psychopathology unit that takes care of parents and children, whose ages vary between 0 and 3 years. This unit receives many migrant mothers.

The population of our study is composed of mother–child dyads in which the migrant mothers have been exposed to traumatic events, in the absence of the infant, before or after its birth. The child's age was between 0 and 3 years. Eligible mothers were informed about the purpose and methodology of the research. Approximately two to three weeks before the interview, they were given the research protocol and the consent form by hand. They were invited to participate in the semi-structured interview in the presence of their baby and to complete the IES-R. A score of 33 calculated with the sum of the scores suggests the presence of post-traumatic stress disorder [16].

### The procedure of the interview

Before the interview began, mothers were given additional verbal information about the research process and context. Each mother participating in the interview provided a written informed consent, which also covered the use of the filmed data. The mothers could refuse to continue the interview at any time.

The semi-structured interview addressed several aspects of the mother–child relationship and the mother's traumatic experience. Each interview lasted on average between 1 and 1 h 30 min. It was conducted in three stages: the first stage concerned the mother's representations of her baby and her relationship with him. In the second stage, the mother was asked to talk about her traumatic experience: the number and nature of the events, temporality, migration, change of situation, loss of family ties, bereavement, etc. The last stage of the interview asked the mother about the presence of the baby's father, the baby's name, the "protective" factors for her and her baby, etc. The question of the mother's narration of the traumatic experience to the child was also asked at the end of the interview. We asked them: “What do you think about sharing, with your child, the traumatic experiences you have had?” From the response to this open question, further questions were addressed to them.

### Analysis strategy

Descriptive statistics were used to analyse quantitative data (i.e., sociodemographic data and IES-R scores) through SPSS v. 22.00. We used a thematic analysis method in order to analyse the corpus of qualitative data we collected. As a matter of fact, the themes explored during our interviews were not systematically defined beforehand. The approach of our analysis is based on six-step gradual and iterative analysis process [17]. First, after interviews were transcribed, each transcript was read with particular focus on first impressions, which were annotated. This is a fundamental step to become familiar with the data [17] but also to develop a deep cross-cultural understanding of the participants' narratives through lexical and semantical nuances. This latter aspect is important while handling cross-cultural samples [18]. The second step consisted in generating initial codes. For this purpose, the data were handled using NVivo 10 for Windows, one of the most used software for qualitative data [19]. Two co-authors (EGN and MEH) worked inductively and created "nodes" in NVivo to reflect "categories" of data observed. At the third step 3, using different functions offered by NVivo, the preliminary codes had been aggregated into broader themes that converged toward a common meaning. In the steps 4 and 5, we reviewed and defined themes, by means of organising and refining into relevant "categories" in order to avoid the overlap between themes, and the dispersion and heterogeneity of the data. At the step 6, finally, each theme was analysed and illustrated by specific quotes. All authors contributed to this phase of the analysis and paid detailed attention to the cross-cultural pattern of participants’ narratives [18]. To ensure anonymity, all quotes that could identify the dyads were concealed without altering the facts.

## Results

### Characteristics of the study group

Twenty mother-baby dyads (n = 20) were contacted to participate in the study. Six dyads refused to participate in the study: five of them because of language barrier and one did not wish to specify the reason for her refusal. In total, our study is based on the analysis of data from fourteen dyads. Table 1 presents the main characteristics of the fourteen dyads. They were migrant women from Africa and Albania. The average age of the participants was 29.9 years; the youngest was 19 and the oldest 42. The majority has been living in France for at least one year. The participants had experienced various traumatic events such as domestic and family violence, excision, rape, war, and difficult migratory paths. The children range in age from 15 days to 30 months. There were seven boys and seven girls. Among the 14 mothers, 4 chose to remain silent about the traumatic event, 7 chose to disclose and the other 3 were hesitant about the choice to disclosure.

**Table 1:** Socio-demographic profile and trauma characteristics of dyads

DYAD	Origin	Age of the mother	Age of the baby (acc. to months)	Gender of the baby	Number of children	Date of arrival in France	Types of trauma	Nature of narrative
Dyad 1	Senegal	26	13	M	1	2016	Domestic and family violences	Choice of disclosure
Dyad 2	Algeria	42	21	F	5	2017	Domestic violences	Choice of disclosure
Dyad 3	Albania	28	30	M	2	2016	Domestic and family violences, difficult migratory journey	Choice of silence
Dyad 4	DRC(Democratic Republic of Congo)	31	15	F	1	2018	Rape, difficult migratory journey	Choice of disclosure
Dyad 5	Mali-Senegal	31	7	F	2	2018	Excision and domestic violences	Hesitation on the choice of disclosure
Dyad 6	Ivory-Coast	33	30	M	4	2016	Rape, Domestic violences, difficult migratory journey	Choice of silence
Dyad 7	Mali	29	15 days	F	2	2019	Domestic violences, excision, difficult migratory journey	Hesitation on the choice of disclosure
Dyad 8	Guinea	23	8	M	2	2017	Domestic violences, forced marriage, difficult migratory journey	Choice of disclosure
Dyad 9	Ivory Coast	19	13	F	1	2018	Forced marriage, difficult migratory journey	Choice of disclosure
Dyad 10	Ivory Coast	29	12	M	1	2018	Domestic violence, difficult migratory journey	Choice of disclosure
Dyad 11	Guinea	26	12	F	2	2017	Excision, domestic violence, difficult migratory journey	Choice of disclosure
Dyad 12	Ivory Coast	34	11	M	3	2016	War, difficult migratory journey	Hesitation on the choice of disclosure
Dyad 13	DRC	35	29	M	5	2017	War, difficult migratory journey	Choice of silence
Dyad 14	DRC	33	20	F	4	2018	Rape	Choice of silence

**Fig. 1:**
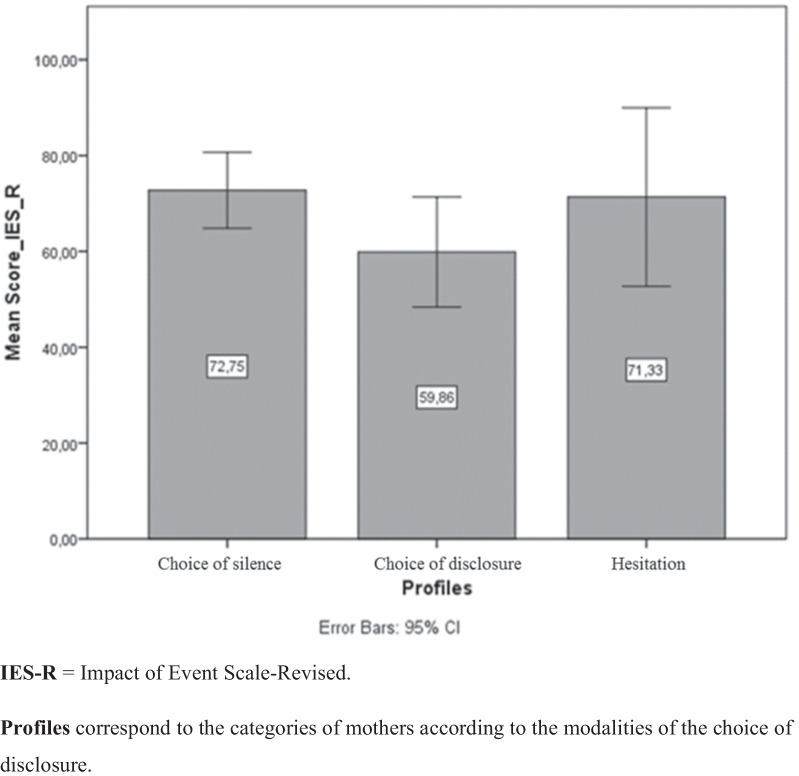
The choice of disclosure according to the intensity of post-traumatic stress symptoms. *IES-R* Impact of Event Scale-Revised. Profiles correspond to the categories of mothers according to the modalities of the choice of disclosure

In the light of the characteristics of the trauma presented in Table 1, we do not observe significant relationship between the types of traumas and the nature of the story, i.e. the choice to tell or to not tell the child the traumatic story. In fact, mothers who chose to remain silent (Dyads 3 and 14) or who were hesitant about the choice to disclose (Dyads 7 and 12) experienced rape, domestic violence, and a difficult migratory journey in the same way as mothers who chose to disclose (Dyads 4 and 8). However, the results show that the choice for disclosure was statistically associated with the severity of the post-traumatic symptoms, F(2, 11) = 4,62, p < 0.05. Specifically, as shown by the Fig. 1, women who made the choice of silence (M = 72.75, SD = 4.99) and those who hesitated on the choice of disclosure (M = 71.33, SD = 7.51) reported higher scores on IES-R than those who made the choice of disclosure (M = 59.86, SD = 12.44).

### Presentation of the discourse analysis

#### Personalization of the traumatic experience

In this theme, the elements highlight the personalization of the traumatic experience in the sense that the mothers appropriate the traumatic story as an experience that is part of their personal life spectrum and does not directly concern their children. They draw a line between their personal experience and the children's story. One of the dyads said:

“It's something in my life that I'm not going to share with my children. For other things I'll say it, but in relation to my life story, no, I'm afraid of their reactions. I don't want to hurt my children. I can tell them that I have not had an easy life but not in detail. No. ”(Dyad 3)

#### The child seen as a weapon against collapse

The mothers' discourse shows that their perceptions of their children are closely linked to the search for their own emotional balance. The project of one day revealing to the child the traumatic story of his conception and birth is seen as an experience that could break this emotional balance. Thus, the child serves as a crutch or aid that protects against the resurgence of painful memories.

“When I wake up, I think, if it wasn't my son, would I be able to get through this? I can lose everything, but my son, I have to have him with me. Sometimes he'd be asleep and I'd need someone to cuddle with, so I'd pick him up and cuddle him and put him back in bed! I've done that so far. So, it helps me a lot. It's very important, it helps us not to think, not to cry, not to get depressed and everything.” (Dyad 1)

Today I can say that it is because of my children that I am well. Otherwise, I don't know. I don't know what would happen but when I think about the children, I try to get up in the morning, I do what I have to do for them. (Dyad 6)

“I can say that if I exist today, it is only for her (pointing to her daughter). I have to be alive for her. I live only for her. She is the one who gives me the taste of life, so if she is not there nothing makes sense. A mother who lives only for her child. ”(Dyad 4)

#### Fear of the child's personal reactions (to the mother, hatred of the father)

The elements in this theme highlight the fear of hostile reactions from the children. Assuming that the children are informed of their history, the mothers fear being brutally confronted with feelings of guilt and disarray linked to their responsibility and powerlessness in the face of the facts. The mothers' discourse also reveals the fear that the children will direct their feelings of hatred and aggression against their father. This comes out explicitly in the discourse of dyad 1.

What I'm afraid of is that she'll be angry with me, that she'll think that I've put them in pain. I'm afraid she'll take it out on me and say that everything she's been through is because of me. (Dyad 6)

I'm afraid to tell him about it and then he'll have a grudge against his father. After all, he needs his father. But maybe he'll want to know why me and his father got divorced. (Dyad 1)

#### Possible partial transmission

It is clear from some mothers' statements that they do not plan to tell their children the whole of their traumatic experience. The part of the story that can be passed on is the part that the mother considers acceptable, less painful, and which may seem to be tolerable for the children.

I can tell them that I have not had an easy life, but not in detail. I can tell them that I have not had an easy life, but not in detail, for other things ok, but not about me, I am afraid of their reactions. (Dyad 3).

#### The trauma narrative according to the child's age

Through this theme, the accounts highlight that one of the mothers' fears is essentially the age at which the story should be revealed to the child. Some mothers think that the story is unbearable for the children, because of their psychological immaturity. Others are hesitant. The hesitation here is in relation to the child's immaturity.

No, I won't tell. No. Because the difficulties I have seen, I don't want to tell the children, because it's very difficult. It's very difficult to tell them at that age that you've slept on the street for several nights, that you've been homeless. How do you tell them? No, no, it's not right. (Dyad 13)

I don't know. I don't know if I'm going to tell them all this. For the moment, no, they are small. (Dyad 7).

The notion of maturity, as a matter of fact, varies depending on the mothers' discourse. For some, this notion is vague, without any temporal delimitation. For others, the age of maturity is delimited from the age of 15. The discourse of these mothers reveals a planned choice for disclosure.

Yes, yes. One day, I'll tell him, especially Bb I, how he came to be here, everything we went through with him. I will explain everything, yes. The day they grow up, yes, I will. (Dyad 8)

Yes, in the future I will tell her. When she is able, 15 or 16, when she wants to go to secondary school or after secondary school, but at least I will prepare her. (Dyad 4)

#### The trap of the cultural in-between: “Rape is not told in our country”

Through this theme, it emerges that mothers are ambivalent about telling their children the story of their conception or birth, and that this ambivalence manifests itself against the background of a cultural conflict. For some mothers, this story is not told in the cultural groups of their countries of origin. However, according to them, the assimilation of the norms and codes of the host country as well as the children's intellectual awakening and curiosity forces them to reveal this story to the children.

Here in France, we say that you have to tell things to children, you have to tell the truth. But hiding things from her for the moment is protecting her. For me it's only to protect her. (Dyad 6)

I don't think so. It's not easy to talk about this kind of thing with children. (Dyad 14).

## Discussion

Based on previous research and identifying a gap in knowledge and understanding around trauma disclosure in migratory context specifically in France, the main objective of this study was to examine the choice of disclosing the traumatic history experienced, in a migratory context, by the mother to the child. Adopting a sequential mixed methods approach, we specifically sought to explore how these mothers projected themselves in relation to the disclosure of traumatic events to their children. Since the children were very young at the time of the study (between 15 days and 30 months), we think that the mothers' statements about trauma disclosure certainly refer to behaviour in the future and not to current behaviour even if it was not explicitly asked. Our findings indicated that the mothers' choice to tell or not to tell their children the story of the trauma did not depend on the type of trauma experienced. However, the results suggested that this choice was influenced by the intensity of post-traumatic symptoms (Fig. 1). Among the categories of women who did not plan to tell their children the story of the trauma and those who were hesitant, several elements motivated this resistance. Through their accounts, we observed that they personalised their traumatic experience and drew a line between this personal experience and the children's story. Among those who appeared to be very vulnerable, the child was seen as a *crutch* that protected them from emotional collapse. In this case, revealing the story would lead to a loss of self-control. Some mothers mentioned the possibility of a partial transmission. However, others were doubtful because of the child's age, who would not be able to bear the traumatic burden of disclosing this story. Additionally, we identified a third group of mothers who took into consideration the socio-cultural factors before deciding to disclose or not the story. It appeared, nevertheless, that the socio-educational environment of the host country forces them to a "form" of psychological transparency that contrasts with the educational practices of the country of origin.

The mothers' choice to tell or not to tell the story of the trauma they experienced did not depend on the type of trauma. In actual fact, the results of our study show that mothers who experienced the same type of trauma had different positions on the choice of disclosure. For example, while one, among the mothers of dyads 4 and 6 who were victims of rape, intended to disclose, the other opted for silence. This result suggests that other factors may be involved in the choice to disclose. These factors may include the subject's personality and parent–child communication patterns. The results of the study by Braga et al. are illuminating in this respect [3]. In their study, although the parents had experienced the same trauma, which was the Holocaust, they had opted for different ways of communicating this trauma to their children. Some had chosen an open, loving and everyday communication style, and yet others had chosen secrets, silence and unspoken words. Our study makes a new contribution by showing that the level of elaboration and psychic integration of the traumatic story also influenced the choice of disclosure. Mothers with more severe post-traumatic symptoms were reluctant or hesitant to share their traumatic experience with their child. Mothers who held this position showed a negative alteration in thymia and cognition marked by sadness of mood, feelings of guilt and shame. For these mothers, "narrating" was not always the most appropriate strategy to protect their children from traumatic transmission [20]. They felt responsible for the events they experienced, such as the rape, the "insecure" environment they would have provided (e.g., lack of fixed accommodation, nights spent on the street without food). The elements of the interview showed that they were in a state of avoidance to narrate these events. There is a kind of psychic negotiation among these mothers about the status of the story, i.e., keeping the story quiet for fear of the child's reaction to the story.

The temporality of disclosure of the traumatic experience to the child is a notion that stands out in our results. Some mothers clearly mentioned the fear of disclosing their painful experience to their young children. They took their child with almost the same qualities of Winnicott's transitional object [21]: the child reassures the mother by its presence, it contains, and protects. And to risk revealing the traumatic story is to appear weak and damaged to a person who perceives you as strong and invincible in the early years. They thought that this story was unbearable for children because of the level of their psychological and emotional maturity. This brings us back to the notion of “modulated disclosure approach,” which consists in telling the traumatic story to the child but following the rhythm of the child's psycho-emotional development [12]. For these mothers, the telling of the trauma story to their children must take into account their personal capacities to integrate the story. In addition, we identified another category of mothers who projected themselves in a partial, and therefore incomplete, disclosure approach. This approach implies a protective attitude towards the children and possibly towards oneself against the unintegrated aspects of the traumatic narrative. We think that this corresponds to the findings of Faundez et al.'s study, where in most cases parents and grandparents, who were victims of political imprisonment and torture during the Chilean military dictatorship, concealed the painful aspects of this experience from their children and grandchildren when they told the story [22]. The memories and emotions surrounding these events were not integrated, either because they were not elaborated enough, or because of the fear that their expression would harm the children and grandchildren.

For other mothers still, there are cultural factors that contrast with the implicit injunction from the host country's socio-educational environment to 'speak out'. Indeed, in many communitarian societies, a pregnant woman who gives birth is in a moment of renewal, of rebirth. She needs something new, all the more so if she is cutting her ties with her former husband. In West and Central Africa, for example, the biological function is not the most important one. Many men and women can play an emotional and educational role with the child through social kinship [23]. In these societies, the disclosure of the traumatic story to the child is not always ensured by the mother; it is mainly ensured by aunts or uncles or grandparents [24]. This chain of transmission limits the traumatic burden associated with disclosure. The migratory context does not offer this environment of the country of origin. The mother finds herself disoriented in relation to her internalised cultural references and the injunction to speak out. Silence then becomes an option in spite of herself, experienced with a feeling of guilt.

## Conclusions and implications

Our study revealed three profiles of mothers with regard to the choice of disclosure of the traumatic story to the child: one group of mothers opted for silence, another for modulated or partial disclosure, and the last group hesitated. We observed that the choice to reveal the story is conditioned by the intensity of the post-traumatic stress symptoms. It appears that when these mothers are still in shock from the weight of the events they have experienced, any prospect of elaborating the trauma story and disclosing it is difficult. In this respect, we can say that it is not the liberation of speech that is most important, but the 'liberation of listening', i.e., in order for the speech to be heard, an ear is needed to receive it. The telling of the traumatic story for the mothers in our study can be facilitated through a caring and supportive therapeutic relationship with the clinicians who receive their story. Our findings suggest that if these mothers recover from their trauma through culturally appropriate therapeutic care, this can effectively contribute to the choice of disclosure and to the development of open and healthy communication strategies to prevent the transmission of their trauma narrative to children. We hope that future studies will be able to examine, with migrant mothers, the psychopathological impact of the modalities of the choice to disclose on the functioning of the mother–child dyad and the communication strategies linked to these modalities. We also think that these modalities of disclosure may evolve with the age of the children. As a matter of fact, it must be stated that the statements of the participants concern much more their intentions at the moment the study was carried out. These statements may change depending on the age of the child and under many conditions, our one time-point study does not capture. Future studies could also take this factor into account to better appreciate the nature of these women's narratives. Finally, our results show that a single theoretical approach is insufficient to understand the motivations underlying the choice to tell or not to tell the traumatic story of mothers to their children. An interdisciplinary, plural and cross-cultural approach would be more appropriate to examine the complexity of the problem of intergenerational transmission of trauma, particularly in a migratory context.

## Data Availability

The datasets generated and/or analysed during the current study are not publicly available due this is what is provided for by the ethical protocol no. 12-065 because these are anonymized but qualitative data. But data are available from the corresponding author on reasonable request.
